# Blocking muscarinic receptors in the olfactory bulb impairs performance on an olfactory short-term memory task

**DOI:** 10.3389/fnbeh.2012.00059

**Published:** 2012-09-06

**Authors:** Sasha Devore, Laura C. Manella, Christiane Linster

**Affiliations:** Computational Physiology Laboratory, Department of Neurobiology and Behavior, Cornell UniversityIthaca, NY, USA

**Keywords:** acetylcholine, scopolamine, delayed match-to-sample, olfactory bulb, olfaction

## Abstract

Cholinergic inputs to cortical processing networks have long been associated with attentional and top-down processing. Experimental and theoretical studies suggest that cholinergic inputs to the main olfactory bulb (OB) can modulate both neural and behavioral odor discrimination. Previous experiments from our laboratory and others demonstrate that blockade of nicotinic receptors directly impairs olfactory discrimination, whereas blockade of muscarinic receptors only measurably impairs olfactory perception when task demands are made more challenging, such as when very low-concentration odors are used or rats are required to maintain sensory memory over long durations. To further investigate the role of muscarinic signaling in the OB, we developed an olfactory delayed match-to-sample task using a digging-based behavioral paradigm. We find that rats are able to maintain robust short-term odor memory for 10–100 s. To investigate the role of muscarinic signaling in task performance, we bilaterally infused scopolamine into the OB. We find that high dosages of scopolamine (38 mM) impair performance on the task across all delays tested, including the baseline condition with no delay, whereas lower dosages (7.6 mM and 22.8 mM) had no measureable effects. These results indicate that general execution of the match-to-sample task, even with no delay, is at least partially dependent on muscarinic signaling in the OB.

## Introduction

Cholinergic inputs to cortical processing networks have long been proposed to be associated with attentional and top-down processing (Hasselmo et al., 1992; Sarter and Bruno, 1997; Sarter et al., 2005; Yu and Dayan, 2005; Hasselmo and Giocomo, 2006). Cholinergic projections originating in the basal forebrain target early sensory processing areas as well as higher-order association and executive processing areas (Mesulam et al., 1983). Because of the strong correlations between sensory inputs, neural activity and perception, we and others have investigated the role of cholinergic inputs for task attention and perceptual discrimination in the main olfactory bulb (OB) of rodents (Linster et al., 2001; Cleland et al., 2002; Linster and Cleland, 2002; Cleland and Linster, 2005). The OB receives extensive cholinergic inputs from the basal forebrain via the nucleus of the horizontal limb of the diagonal band of Broca (HDB) that innervates primarily glomerular and granule cell layers of the bulb (Heimer et al., 1990). Recent experiments from our laboratory suggest that while the cholinergic inputs to the OB can modulate perceptual discrimination between odorants (Linster and Cleland, 2002; Mandairon et al., 2006; Chaudhury et al., 2009) they do not seem to affect the acquisition of an odor memory or an odor-reward association *per se* (Linster and Cleland, 2002; Mandairon et al., 2006). In particular, the formation of a non-associative odor memory was not affected by local bulbar manipulations of cholinergic function; however, the specificity of this memory was increased when ACh was enhanced and decreased when ACh was blocked (Hunter and Murray, 1989; Mandairon et al., 2006). Similarly, when rats were trained to associate an odor with a food reward, the specificity but not the strength of this association was modulated by cholinergic inputs, both when immunotoxic lesions of cholinergic neurons (Linster et al., 2001) or local infusions of cholinergic antagonists were used (Chaudhury et al., 2009).

Although acetylcholine in the OB acts on both nicotinic and muscarinic receptors (Castillo et al., 1999; Ghatpande et al., 2006; Pressler et al., 2007), results from these previous studies have suggested a critical role for nicotinic cholinergic receptors in odor discrimination, whereas muscarinic receptors seemed to play a secondary role: blockade of muscarinic receptors, while not producing significant effects by itself, potentiated the observed effects of nicotinic receptor blockade (Mandairon et al., 2006; Chaudhury et al., 2009). However, studies from other laboratories suggest a direct role for muscarinic receptors in a more challenging associative olfactory short-term memory task (Ravel et al., 1994). Here, we further investigate the role of muscarinic receptor modulation in OB processing by adapting an olfactory short-term memory task—delayed match-to-sample (Ravel et al., 1994)—our digging-based behavioral paradigm (Cleland et al., 2002). We find that blocking muscarinic receptors leads to a decrease in task performance independent of the time delay over which rats are required to maintain short-term memory. These results indicate a direct functional role for muscarinic receptors in regulating olfactory processing that may extend beyond short-term memory, *per se*.

## Materials and methods

### Subjects

Six adult male Long–Evans rats initially weighing 250–300 g were obtained from Charles River Laboratory (Wilmington, MA). Rats were housed individually in standard laboratory cages on a 12-h reversed light/dark cycle (lights on at 21:00 h), with behavioral testing taking place during the dark hours. Rats were given access to water *ad libitum* but were maintained on a food-deprivation schedule to keep them 85–90% of their free-feeding body weight over the course of behavioral testing. All procedures were approved by the Cornell University Institutional Animal Care and Use Committee.

### Odorants

For all experiments, we used a set of 12 structurally unrelated odorants (Table 1). Odors were diluted in mineral oil so as to theoretically emit a steady-state vapor-phase partial pressure of 3 Pa. Odorized bedding was made by mixing 1.2 mL of odor with 1 L of small-kernel corncob bedding (Bed-O-Cobs, The Andersons, Maumee, OH). Odorized bedding was mixed twice per week and stored in air-tight plastic containers.

**Table 1:** **Odors used in Experiments 1–3 with corresponding percentage v/v dilution**.

**Odor name**	**% v/v Dilution**
Hexanoic Acid	4.463
Butanol	0.062
Butyl Acetate	0.065
Isoamyl Acetate	0.151
2-octanone	0.524
Pentyl Butyrate	1.715
(+)-Limonene	0.612
Heptanal	0.212
Citronellal	4.975
(1, 8)-Cineole	0.586
Anisole	0.155
(+)-Carvone	14.147

### Behavioral training

All behavioral training and testing took place in a transparent cage (46 × 25 × 22 cm) fitted with an opaque divider. Rats were acclimated to the experimental apparatus over the course of several days and then shaped to dig for cereal rewards (Malt-o-Meal Fruity Loops), pre-exposed to heated air (93°C) to reduce odor, buried in a ceramic dish (9 cm diameter, 4.5 cm depth) containing unscented bedding.

**Figure 1:**
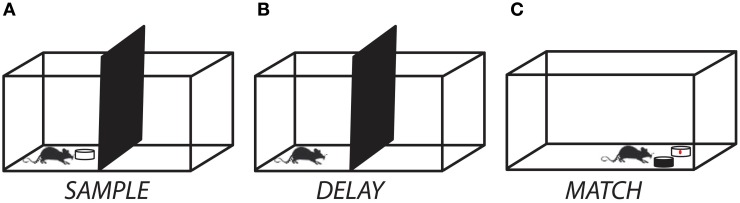
**Experimental setup for the delayed match-to-sample task. (A)** At the start of a trial, the rat was presented with a ceramic dish containing odorized bedding. **(B)** After allowing for 10–15 s of investigation, the sample odor dish was removed and a delay period (ranging from 0 s to 10 min) was imposed. **(C)** At the end of the delay period, the divider was raised and the rat moved to the test chamber and had to discriminate between a dish containing the matching odor (rewarded) and one containing a non-matching odor (unrewarded). A trial was counted as correct if the rat initiated digging in the matching odor dish first.

Rats were then shaped incrementally to perform the match-to-sample task. First, rats were trained to investigate a dish containing odorized bedding (“*sample*”) that was placed on the start side of the testing chamber (Figure 1A). After allowing for approximately 8–10 s of active investigation, the *sample* was removed and the divider lifted, giving the rat access to an identical dish of odorized bedding placed on the opposite side of the testing chamber (“*match*”). The rat was trained to dig in the *match* for a cereal reward. Next, a non-rewarded dish (“*non-match*”) containing unscented bedding was placed next to the *match* and rats were trained to selectively dig in the *match* for the cereal reward. In the third and final stage of training, the non-rewarded odor dish was filled with scented bedding of a different odor than the *sample/match* and the rats were trained to selectively dig for the cereal reward in the *match* while avoiding the *non-match* (Figure 1C). The *match* and *non-match* dishes were always placed in a random orientation, so that odor was the only reliable cue for reward. For each training session, each of the 12 odorants (Table 1) served as both *sample/match* and *non-match*, pseudo-randomly ordered such that no odor appeared on consecutive trials. We allowed rats to self-correct only during the intermediate but not the final training phase. Training was complete when rats reached criterion performance of at least 10 correct responses out of 12 trials for two consecutive sessions. Following training, rats were prepared for surgical implantation of cannula.

### Surgery

Rats were anesthetized using an intramuscular injection of a mixture of ketamine/xylazine (50 mg/kg ketamine, 7.5 mg/kg xylazine, injection volume 1 mL/kg) and then secured on a stereotaxic device (Narishige Instruments, Tokyo, Japan). Guide cannulae (22-gauge; Plastics One, Roanoke, VA, USA) were implanted bilaterally into the OBs (AP, +8 mm, ML, ±1.9 mm, DV, −4.5 mm) and affixed to the skull with stainless steel bone screws and dental cement. The tips of the guide cannulae were positioned 1 mm dorsal to the target infusion site, with infusion needles extending 1 mm beyond the tip. Dummy cannulae were used to prevent blockage or infection. Rats recovered for at least ten days after surgery and were then retrained on the match-to-sample task (see above) until they reached criterion performance.

### Experiment 1: delayed match-to-sample task

Experiment 1 established the time course of olfactory short-term memory in our match-to-sample paradigm. In this experiment, a time delay was imposed between removal of the *sample* and lifting of the barrier (Figure 1B). On each of four testing days, rats completed five trials with 0 s delay and then performed one test trial at each of six delays (0, 30, 60, 120, 300, 600 s) randomly ordered, with at least 300 s between trials. In principle, the 0 s delay condition corresponds to an immediate memory test, but in practice there was an approximately 2 s motor delay due to the experimenter lifting the barrier and the rat traversing the chamber. For each testing session, the 12 odorants (Table 1) were pseudo-randomly assigned such that they appeared as *sample/match* and *non-match* no more than once and never on consecutive trials.

### Experiment 2: reward-detection control task

Rats performed a single session of a reward-detection control task to determine if they were able to use cues other than the test odors in the delayed match-to-sample experiment. The control task consisted of the same match-to-sample paradigm as in Experiment 1, except the *sample*, *match*, and *non-match* contained identical odors. Rats performed one trial with 0 s delay for each of the 12 odors in the odor battery (Table 1), randomly ordered, with at least 300 s between trials.

### Experiment 3: effects of muscarinic antagonists on short-term memory

#### Pharmacology

Rats were tested using four drug conditions: the selective muscarinic receptor antagonist scopolamine hydrobromide (Sigma Aldrich, St. Louis, MO) at three dosages (7.6, 22.8, or 38 mM) and 0.9% sterile saline as a control. Scopolamine was prepared weekly by dissolving in 0.9% sterile saline and stored in small aliquots for daily use. Before each experimental session, animals received bilateral infusions of either scopolamine or vehicle at a rate of 2 μl/min for a total infusion volume of 6 μl per side. The infusion cannulae remained in place for at least 1 min after the infusion ended to prevent backflow. Behavioral testing began 20 min after drug administration was complete. The infusion volumes and drug dosages used in the present study were determined based on previous studies in which cholinergic antagonists were infused into the OB (Mandairon et al., 2006; Chaudhury et al., 2009).

#### Behavioral testing

This experiment was identical to the delayed match-to-sample task (Experiment 1, above), except that rats were tested only on delays of 0 and 120 s, corresponding to baseline and short-term memory tests, respectively. In each session, rats performed two trials with 0 s delay followed by four trials each with 0 and 120 s delay, randomly ordered, with at least 300 s between trials. For each session, the 12 odorants (Table 1) were pseudo-randomly assigned such that they appeared as *sample/match* and *non-match* no more than once and never on consecutive trials. Rats completed experimental sessions with drug infusions every second day interleaved with a retraining session in which all trials had 0 s delay. Rats completed at least three experimental sessions for each of the four drug conditions.

### Data analysis

Data analysis was performed using SPSS statistical software (SPSS, Chicago, IL). Performance was assessed by computing the fraction of correct trials at each delay. Repeated measures analyses of variance (ANOVAs) were used to test for a significant effect of delay (Experiments 1 and 3) or drug and delay (Experiment 2). Fisher's *post-hoc* pairwise comparisons were used to determine the significance of differences between specific delays or drug conditions. The criterion for significance was set at α = 0.05.

### Histology

**Figure 2:**
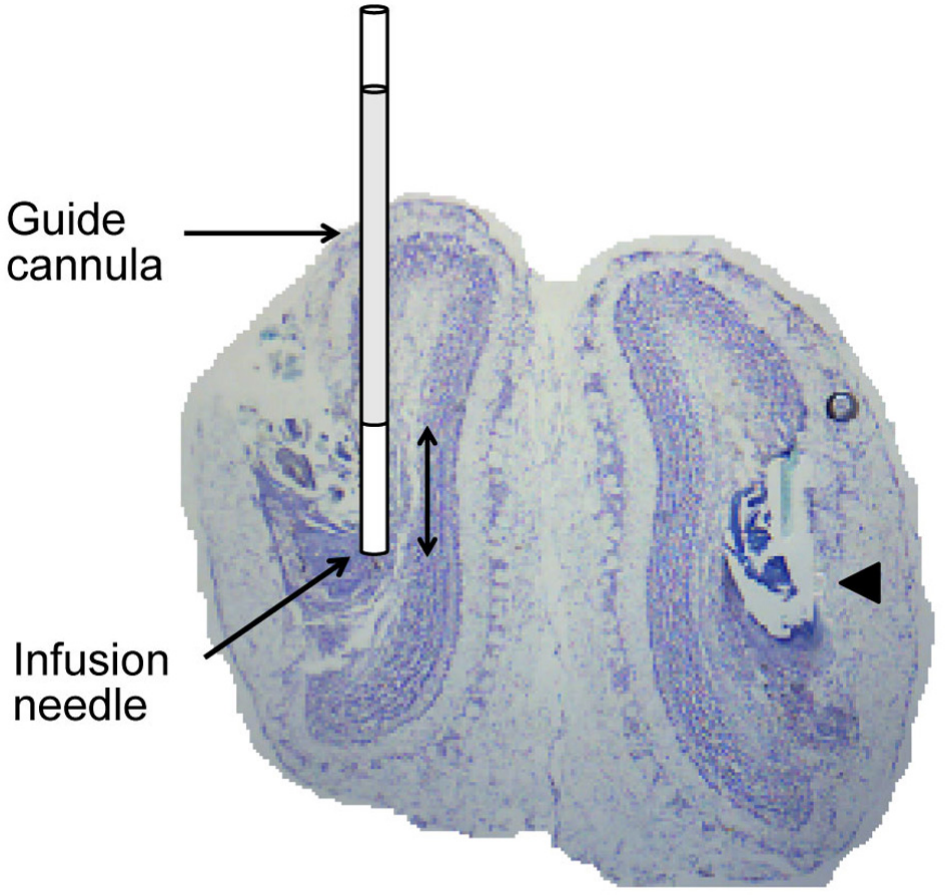
**Histological verification of cannula placement.** Coronal section through the OB illustrating placement of guide cannula and infusion needle. The arrowhead points to a cannula track in the right OB. Double headed arrow is 1 mm.

After completing all behavioral sessions, rats were deeply anesthetized with an intraperitoneal injection of urethane (1.5 mg/kg) and received bilateral infusions of 1% methylene blue (6 uL per side). 20 min after the infusion was complete, rats were sacrificed by cardiac perfusion of 0.9% saline followed by 10% formalin. The brain was extracted and visually inspected to confirm methylene blue diffusion within the main OBs. The brain was soaked in 10% formalin solution for several days, saturated in a solution of 20% sucrose in PBS and then sectioned in 40 μm slices and stained with cresyl violet in order to verify the location of cannula tracts (Figure 2).

## Results

With moderate training, all animals (*n* = 6) were able to perform the match-to-sample digging task to criterion, defined as >80% correct across two consecutive experimental sessions. Rats took an average of 113 ± 33 trials to reach criterion performance, corresponding to an average of 10 training sessions.

**Figure 3:**
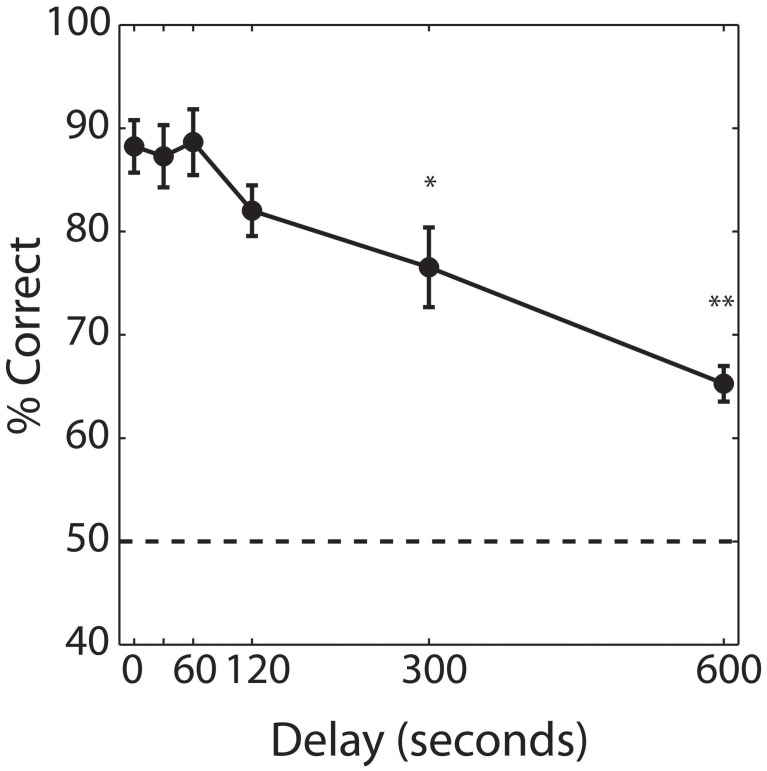
**Performance on the baseline delayed match-to-sample.** Percent correct performance as a function of delay, averaged across subjects (*n* = 6). Error bars indicate ±1 SEM. Asterisks indicates significant differences of ^*^P < 0.05 and ^**^P < 0.001 from performance obtained with a 0 s delay (baseline memory test).

Our first aim was to determine the time course of olfactory short-term memory in the delayed match-to-sample task (Experiment 1). Figure 3 shows performance as a function of delay, averaged across subjects. Performance in the baseline condition, with 0 s delay, averaged 88.3% (±7.3%). Rats maintained ceiling performance for time delays of 30 and 60 s, with accuracy declining at delays greater than 120 s. A repeated measures ANOVA yielded a significant effect of delay [*F*_(5, 42)_ = 3.852, *P* = 0.001] with *post-hoc* comparisons indicating that performance was significantly worse than baseline at delays of 5 min (*P* < 0.05) and 10 min (*P* < 0.001). However, even at 10 min, the longest delay at which we tested short-term memory, performance averaged 65.7% (±4.9%) and was significantly greater than chance (*P* < 0.001).

To ensure that rats' weren't using residual odor cues from the buried cereal reward to solve the task, we ran a control experiment (Experiment 2) in which all odors—*sample, match, and non-match*—were identical. Average performance on this task was 48.39% (±10.4%) and was not significantly different from chance (*P* = 0.785), suggesting that rats are restricted to cues from the test odorants when solving the match-to-sample task.

**Figure 4:**
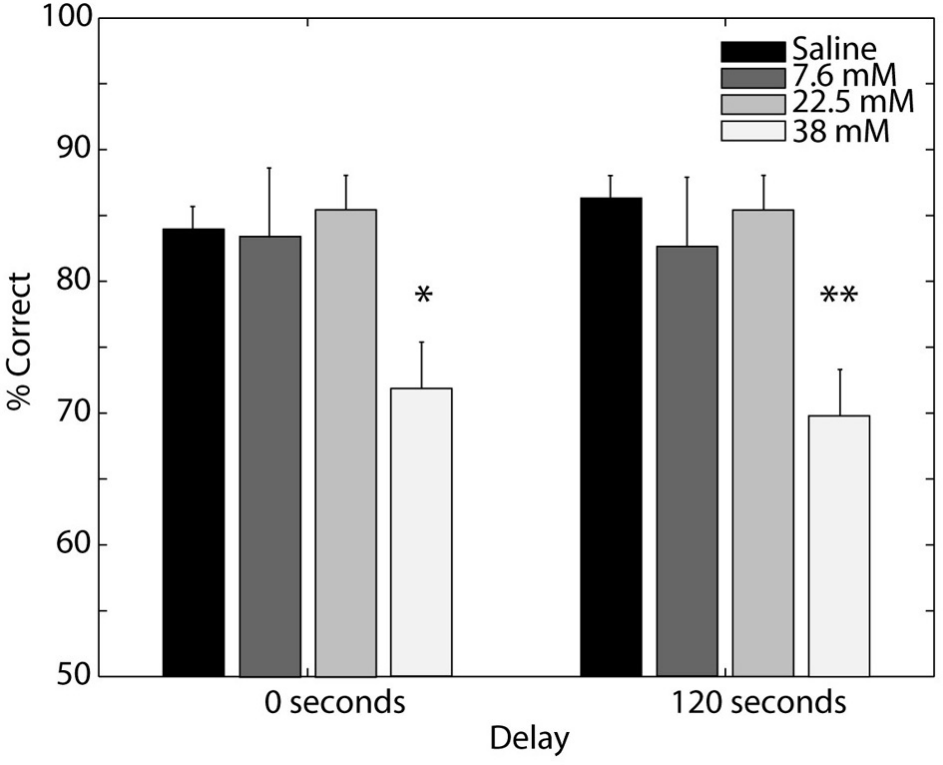
**Effect of blocking bulbar muscarinic receptors on delayed match-to-sample.** Percent correct performance for the baseline (0 s delay) and short-term (120 s delay) memory tests after saline or scopolamine (7.6, 22.5, and 38 mM) infusion into the olfactory bulb. Error bars indicate ±1 SEM. Asterisks indicate significant differences of ^*^P < 0.05 and ^**^P < 0.005 relative to saline controls.

The first experiment established that odor memory in our digging-based delayed match-to-sample task persists for 10–100 s. Previous studies have suggested a role for OB muscarinic receptors in olfactory short-term memory at this timescale (Ravel et al., 1994). Thus, we next sought to determine whether performance on our task requires muscarinic cholinergic receptors in the OB (Experiment 3). We tested the effects of scopolamine infusions into the OB at multiple dosages on rats' ability to perform the delayed match-to-sample task at both short and long delays. In order to obtain a sufficient number of trials at each delay and dosage, we restricted the delays tested to 0 s (baseline) and 120 s. We chose the 120 s delay for the short-term memory test as the slope of the performance-delay curve steepened considerably at this point (Experiment 1). Figure 4 shows the average performance, across subjects, in each of the drug conditions for both the baseline test (solid line) and short-term memory test (dashed line). We found that performance deteriorated with increasing scopolamine concentration, with similar effects at both short and long delays. These trends were confirmed by a Two-Way ANOVA with repeated measures, which yielded a significant effect of drug [*F*_(3, 20)_ = 9.235, *p* = 0.001] but not delay (*P* = 0.940), with no significant interaction (*P* = 0.578). *Post-hoc* comparisons revealed that performance on the task was unaffected by scopolamine at low (7.6 mM) and intermediate (22.8 mM) dosages and decreased significantly at the highest dosage of scopolamine tested (38 mM) on both the baseline (*P* = 0.042) and short-term memory tests (*P* = 0.004). These results demonstrate that cholinergic signaling at muscarinic receptors in the OB is necessary for general task performance, including the baseline condition with no delay between odor sampling and memory test.

## Discussion

The present experiments were designed to test the role of muscarinic signaling in the OB on olfactory short-term memory. We found that high dosages of scopolamine lead to a decline in task performance even in the baseline condition, with minimal delay between odor sampling and memory test. These results suggest that olfactory short-term memory *per se* is not necessarily dependent on muscarinic signaling in the OB; rather, our findings suggest a primary role for muscarinic receptors in the OB in regulating more general aspects of olfactory sensory processing.

The present findings contradict results from a previous study by Ravel et al. (1994), which found a delay-dependent effect of scopolamine on a similar task. However, striking methodological differences preclude a direct comparison between the two studies. In particular, Ravel et al. used only two odors in their study, one of which was randomly assigned as the target odor for each trial. Consequently, their task was more susceptible to proactive interference, which has been shown to be enhanced by blockade of muscarinic receptors (De Rosa and Hasselmo, 2000). In the current study, we used a much larger battery of odors and ensured that rats never encountered the same odors on consecutive trials, thus reducing the possible buildup of proactive interference across trials. In contrast to the Ravel et al. (1994) study, which suggested a direct role for muscarinic receptors in olfactory short-term memory, our study suggests a more general role for muscarinic receptors in the task.

In previous experiments from our laboratory, the same high dosage of scopolamine (38 mM) has typically played a secondary role in olfactory discrimination behavior. Namely, blockade of muscarinic receptors potentiated the effects of nicotinic receptor blockade on spontaneous discrimination of chemically related odorants (Mandairon et al., 2006). On the other hand, neither reward-motivated discrimination of chemically related odorants nor spontaneous discrimination of chemically unrelated odorants was affected by muscarinic or combined muscarinic and nicotinic receptor blockade (Mandairon et al., 2006). In the present experiment, the pairs of odorants presented to the rats were chosen to be perceptually dissimilar (i.e., chemically unrelated); however, the nature of the task itself was difficult. Rats had to remember an odor that was briefly presented in order to make a subsequent choice between this same odor and a novel odor. Additionally, we used a battery of 12 odors randomly assigned into odor pairs for each trial. The task design did not allow for rote learning of the odor pairs, and hence the task was difficult even at zero or very short delays.

In general, all data so far suggest that blockade of muscarinic receptors in the OB measurably impairs olfactory function only when the animals' task is made difficult. Difficulty can be manipulated along multiple dimensions, either by choosing highly similar odorants (Linster et al., 2001; Chaudhury et al., 2009), impairing the system via additional blockade of nicotinic receptors (Mandairon et al., 2006), or by using a short-term memory task with a large odor battery (present “Results”). This idea supports an attentional function for muscarinic modulation in the OB, similar to that proposed in other systems (Hasselmo et al., 1992; Sarter and Bruno, 1997; Sarter et al., 2005; Yu and Dayan, 2005; Hasselmo and Giocomo, 2006).

Cholinergic modulation acting on muscarinic receptors in the OB mainly modulates granule cells, a class of inhibitory neurons located in the external plexiform layer. Acetylcholine leads to changes in granule cell function that can affect the timing and precision of spikes in mitral cells, the principle output neurons from the OB (Pressler et al., 2007; David et al., 2009). In turn, mitral cell spike timing affects postsynaptic processing in the olfactory cortex including spike-timing dependent plasticity (Linster and Cleland, 2010). With muscarinic receptors blocked, the reduced spike timing precision in mitral cell outputs would result in less-selective olfactory cortical odor representations (Linster and Cleland, 2010), which we speculate underlies the task deficits we observed with high dosages of scopolamine. Performance when task demands are low, such as when only two are odors are to be discriminated (Mandairon et al., 2006), may not explicitly depend on selectivity of odor representations. However, as the task becomes increasingly difficult such as when an increasing number of odors are to be discriminated or odors become increasingly similar, selectivity becomes essential.

In summary, previous studies from our laboratory have demonstrated a direct role for nicotinic receptors in regulating the extent of mitral cell odor receptor fields and odor discrimination (Chaudhury et al., 2009). The present results suggest that muscarinic receptors also play a primary role in olfactory sensory processing that may only be unmasked during performance of difficult olfactory tasks. A key question that remains is to what extent the activation of cholinergic inputs to the olfactory system are themselves modulated by task demands and attentional load.

### Conflict of interest statement

The authors declare that the research was conducted in the absence of any commercial or financial relationships that could be construed as a potential conflict of interest.
